# Long‐term oncological outcomes after multimodal treatment for locally advanced prostate cancer

**DOI:** 10.1002/bco2.414

**Published:** 2024-08-01

**Authors:** Fiorella L. Roldan, Ugo Giovanni Falagario, Mats Olsson, Rodolfo Sánchez Salas, Markus Aly, Lars Egevad, Anna Lantz, Henrik Grönberg, Olof Akre, Abolfazl Hosseini, N. Peter Wiklund

**Affiliations:** ^1^ Servei d'Urologia, Hospital Clínic de Barcelona Barcelona Spain; ^2^ Section of Urology, Department of Molecular Medicine and Surgery Karolinska Institute Stockholm Sweden; ^3^ Department of Urology and organ transplantation University of Foggia Foggia Italy; ^4^ Department of Medical Epidemiology and Biostatistics Karolinska Institute Stockholm Sweden; ^5^ Department of Oncology‐Pathology Karolinska Institute Stockholm Sweden; ^6^ Department of Urology Danderyds Hospital Stockholm Sweden; ^7^ Department of Urology University Hospital Basel Switzerland; ^8^ Department of Urology Icahn School of Medicine, Mount Sinai Health System New York New York USA

**Keywords:** cancer‐specific survival, cystoprostatectomy, locally advanced prostate cancer, radical pelvic surgery, radiotherapy

## Abstract

**Objective:**

The aim of this study is to evaluate treatment patterns and long‐term oncological outcomes of patients with locally advanced prostate cancer (LAPCa).

**Patients and methods:**

This is a population‐based study including LAPC (cT3‐4, M0) patients from the Stockholm region (Sweden). A sub‐analysis was performed in men treated with primary cystoprostatectomy or total pelvic exenteration (TPE) for cT4 prostate cancer (PCa).

Cox regression was used to identify predictors of overall mortality (OM) and cancer‐specific mortality (CSM). Biochemical progression‐free survival (BPFS) and 90 days complications were reported for the radical surgery subgroup.

**Results:**

We included 2921 patients with cT3(*N* = 2713) or cT4(*N* = 208), M0 PCa diagnosed between 2003 and 2019. Out of these, 249(9%), 1497(51%) and 1175(40%) underwent radical prostatectomy, RT + ADT and androgen deprivation therapy (ADT), respectively. Survival rates were 76% (IQR: 68, 83), 47% (IQR: 44, 50) and 23% (IQR: 20, 27), respectively at 10 years. Irrespective of treatment modalities, cT4 patients had worse survival compared to cT3 patients (OM: HR1.44, IQR:1.17,1.77; PCSM: HR1.39, IQR:1.06,1.82). Twenty‐seven patients with cT4, N0‐1, M0 were treated with cystoprostatectomy or TPE. Twenty‐two patients (81.5%) received neoadjuvant ADT. The 5‐year BPFS, CSS and OS rates were 39.6%, 68.8% and 63.8%, respectively. Nine patients (33.3%) had Clavien‐Dindo grade III and 1 (3.7%) grade IV complication within 90 days after surgery.

**Conclusions:**

Pelvic surgery with radical intent as part of a multidisciplinary management may be an effective alternative for selected patients with locally advanced PCa leading to local tumour control and an acceptable morbidity.

## 1 INTRODUCTION

Prostate cancer (PCa) is the second most diagnosed cancer among men and remains an important cause of cancer death.^1^ To date, between 5% and 15% of patients are diagnosed with non‐metastatic locally advanced prostate cancer (LAPCa)^2^ defined by the European Association of Urology (EAU) guideline as cT3‐T4 PCa or positive lymph nodes.^3^ LAPCa is associated with metastasis progression, poor cancer‐specific and overall survival.^3^, ^4^

In the absence of high‐level evidence, a recent systematic review could not define the best treatment option for patients with LAPCa.^5^ Randomized controlled trials are only available for long‐term androgen deprivation therapy (ADT) combined with local external beam radiation therapy^3^ showing that local treatment combined with a systemic treatment offers the best outcome. Radical prostatectomy (RP) is suggested for selected patients as part of multimodal therapy.^3^ However, the comparative oncological effectiveness of RP and RT will remain unknown until the results of the SPCG15 trial comparing RP with or without adjuvant or salvage RT against primary RT and ADT.^6^ Besides, LAPCa is a complex entity, including different scenarios as bladder neck involvement or invasion of surrounding pelvic structures, hardly fully eradicated with a prostatectomy and positive margins may have detrimental effects on the oncological outcomes.

Cystoprostatectomy or total pelvic exenteration (TPE) may play a role in the treatment of this subset of patients, initially considered unfit for RP due to the disease extension. It has been described that such surgical strategy may significantly decrease the risk of positive margins, achieve local tumour control, alleviate local urinary symptoms and improve quality of life (QoL).^7^ A systematic review by Yuan et al.,^8^ including primary cystoprostatectomy as the main treatment for cT3‐4 patients N0‐1, considered it as an alternative to the surgical step of the multimodal therapy as it may improve survival outcomes when combined with adjuvant therapies.

We have considered a similar approach, aiming to elucidate whether a more radical surgery could benefit the patient and delay or avoid adjuvant treatments.

The aim of this study was first, to evaluate treatment patterns and long‐term oncological outcomes of patients diagnosed with non‐metastatic LAPCa in a population‐based database and second, report outcomes of surgical treatment with radical intent for the most challenging scenario, namely, clinical T4 PCa treated with primary cystoprostatectomy or TPE.

## 2 MATERIAL AND METHODS

### 2.1 Study populations

The present study leverage analysis on two cohorts: first, the main cohort using data from the Stockholm PSA and Prostate Biopsy register, a population‐based database from Stockholm region to report treatment modalities patterns and corresponding oncological outcomes of patients with non‐metastatic LAPCa. Second, the subgroup where we reported treatment protocols, outcomes and complications of clinical T4 PCa treated with pelvic surgery with radical intent at Karolinska University Hospital.

#### 2.1.1 Main cohort

All patients diagnosed with non‐metastatic local advanced prostate cancer (cT3‐4,M0) in the Stockholm PSA and Prostate Biopsy registry during year 2000 to 2019 were included in this study cohort. The register is linked to the National Prostate Cancer Registry (1996–2019), National Patient Registry, National Prescribed Drug Registry, Cause of Death Registry, National Cancer Registry and the LISA Registry (for information on migration, marital status, income and educational level). A regional ethics board approved the study (Regional Ethics Testing Board, Stockholm; EPN DnR 2012/438‐31/3). After treatment, all patients who continued follow‐up in the Stockholm area received a PSA every 3 months for the first 2 years and every 6 months after. PSA tests were performed in only three centralized laboratories using the latest standards of quality. Imaging was performed at discretion of the treating physician.

#### 2.1.2 Radical surgery subgroup

Our institutional PCa database was used to extract data on patients who underwent cystoprostatectomy or TPE between May 2007 and January 2020. Included patients met the following criteria: cT4 primary PCa, no concomitant malignancies, no distant metastasis, good general performance status with ECOG score 0 to 1 and minimum follow‐up time of 1 year. Clinical positive lymph node disease in the pelvic area including mesorectum nodes was not exclusion criteria. In all patients, bone scan, magnetic resonance imaging (MRI) and abdominal computerized tomography (CT) were used for staging. PSMA PET‐CT scan was performed to dismiss metastasis in case of doubt since 2017. Surgery indication and neoadjuvant treatment (NT) such as hormone therapy, radiotherapy, chemotherapy or any combination were planned in our PCa multidisciplinary conference. If deemed appropriate, neoadjuvant or adjuvant therapies consisted of either one or a combination of complete androgen blockage, neoadjuvant radiotherapy (RT) with five sessions 5Gy each (final dose 25Gy) and neoadjuvant chemotherapy with 4 to 6 doses of Docetaxel. New PSA and imaging evaluation were performed within a week before surgery in patients who received NT. Patients' clinical characteristics, treatment modalities and pathological findings were recorded. All the specimens were totally embedded. After surgery, all patients discontinued the ADT. During the follow‐up period, PSA was regularly tested, and restaging was performed with bone and CT scans at biochemical or clinical progression. Salvage treatments were provided in patients with biochemical or clinical progression according to institutional protocols.

### 2.2 Outcomes definition and statistical analysis

Primary outcome of this study was overall mortality (OM). Secondary outcome was cancer‐specific mortality (CSM).

First, descriptive statistics of the population‐based database was performed to describe preferred treatment modalities for patients with non‐metastatic LAPCa. Survival rates were estimated using the Kaplan–Meier method for each treatment modality and according to clinical stage (cT3 vs. cT4).

Second, we performed multivariable Cox regression analysis in the population‐based registry to determine clinicopathological factors predicting the outcomes of interest. Input parameters were age, Charlson comorbidity index (CCI), PSA at diagnosis, clinical T and N stage, ISUP grade at diagnosis, primary and Salvage treatment modalities.

Finally, we described the Karolinska institute surgical cohort of patients with non‐metastatic LAPCa and cT4 disease in terms of oncological outcomes (OM and CSM), Biochemical progression‐free survival (BPFS, PSA ≥ 0,2 ng/mL in two consecutive measurements), clinical progression‐free survival (local tumour recurrence or distant metastases), radiological response to NT (any tumour volume reduction visible in the MRI) and 90 days complications according to the Clavien‐Dindo classification.

STATA 16.1 was used to conduct data management and analysis of the data.

## 3 RESULTS

### 3.1 Main cohort

Table 1 shows the baseline characteristics of the 2921 cT3/4, M0 patients extracted from the population‐based registry. Out of these, 1497 (51%) and 249 (9%), were treated with RT + ADT and RP, respectively; 1175 (40%) patients did not receive any form of local treatment with curative intent and were managed with ADT only.

**TABLE 1:** Preoperative patients' characteristics and treatment modalities of patients diagnosed with non‐metastatic locally advanced prostate cancer in the Stockholm region.

	Radical prostatectomy (*N* = 249)	Radiotherapy (*N* = 1497)	Non‐curative intent (*N* = 1175)	*p*‐Value
Age (year)	67 (62, 73)	69 (63, 74)	77 (71, 82)	<0.0001
Charlson CI, *n* (%)				
0–1	92 (37%)	77 (5%)	652 (55%)	<0.0001
2	90 (36%)	708 (47%)	121 (10%)	
3+	67 (27%)	712 (48%)	402 (34%)	
RP ISUP, *n* (%)				
1	23 (9.5%)	130 (9.8%)	59 (6.1%)	<0.0001
2	69 (28.5%)	287 (21.6%)	138 (14.3%)	
3	63 (26.0%)	338 (25.5%)	226 (23.4%)	
4	27 (11.2%)	271 (20.4%)	201 (20.8%)	
5	60 (24.8%)	302 (22.7%)	343 (35.5%)	
PSA, ng/mL	12.9 (6.6, 27.0)	20.0 (9.0, 47.0)	34.0 (14.1, 91.0)	<0.0001
cT stage, *n* (%)				
cT3	244 (98.0%)	1408 (94.1%)	1061 (90.3%)	<0.0001
cT4	5 (2.0%)	89 (5.9%)	114 (9.7%)	
cN stage, *n* (%)				
N0	78 (31.6%)	358 (24.1%)	94 (8.0%)	<0.0001
N1	10 (4.0%)	116 (7.8%)	60 (5.1%)	
NX	159 (64.4%)	1012 (68.1%)	1015 (86.8%)	
Salvage radiotherapy, *n* (%)				
No	199 (79.9%)	N/A	N/A	
Yes	50 (20.1%)	N/A	N/A	
Neoadjuvant ADT, *n* (%)				
No	230 (92.4%)	N/A	N/A	
Yes	19 (7.6%)	N/A	N/A	
Adjuvant ADT, *n* (%)				
No	84 (33.7%)	31 (2.1%)	0 (0.0%)	<0.0001
Yes	165 (66.3%)	1466 (97.9%)	1175 (100.0%)	

Abbreviation: ADT, androgen‐deprivation therapy; RP, Radical prostatectomy.

In patients who underwent RP as initial treatment modality, ADT was prescribed in 165 (66%) of the patients, and 50 (20%) patients underwent salvage radiotherapy.

During the follow‐up, 1458 deaths and 746 PCSM occurred. The median follow‐up time of survivors was 8 (6,11) years.

Men treated with RT + ADT or ADT alone were older, with more comorbidities, had higher PSA values and ISUP GG at diagnosis and higher clinical T and N stage. Notably, out of 208 patients with cT4 disease, only 5 (2.4%) underwent RP, and the majority (*n* = 114, 55%) underwent treatment with ADT alone.

**FIGURE 1:**
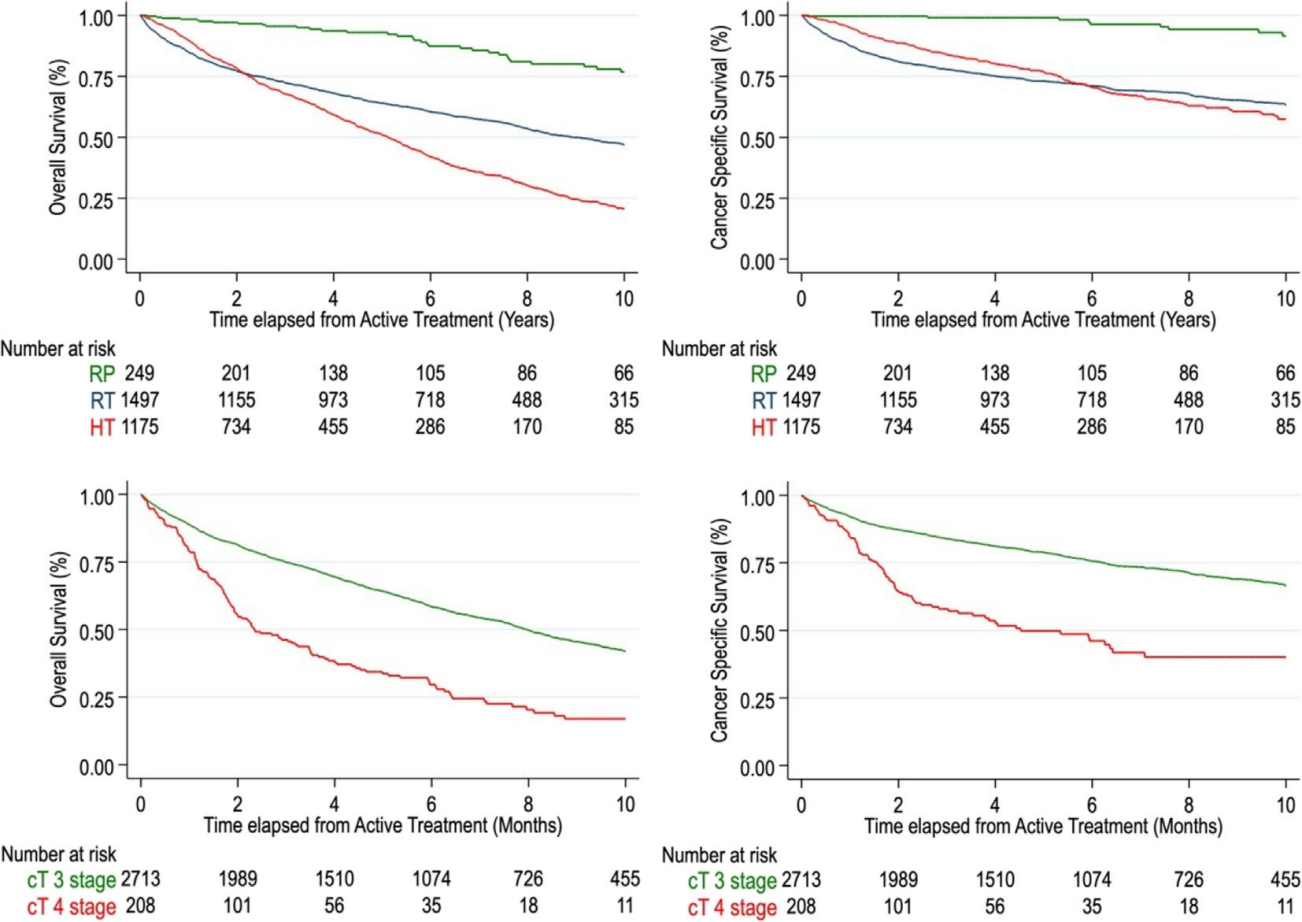
Overall survival and cancer‐specific survival of patients diagnosed with non‐metastatic locally advanced prostate cancer in the Stockholm region.

Kaplan–Meier survival curves are presented in Figure 1; 10 years overall survival rates were 76% (IQR: 68, 83), 47% (IQR: 44, 50) and 23% (IQR: 20, 27) for patients undergoing RP, RT and treatment with ADT alone. Corresponding prostate cancer‐specific survival rates were 92% (IQR: 85, 96), 63% (IQR: 60, 66) and 59% (IQR: 54, 63). At multivariable Cox regression analysis, Age, PSA, CCI, cT 4 disease, cN, bx ISUP GG 4–5 and initial treatment modality were predictors of OM and PCSM (Table S1). Specifically, for OM, the adjusted model showed higher hazard of mortality following RT + ADT or ADT alone in comparison to RP (RT: HR 3.79, IQR: 2.50, 5.97; ADT alone: HR:3.15, IQR:1.99, 4.96). For PCSM, the adjusted HRs in comparison to RP were 7.32 (IQR: 3.00, 17.97) for RT + ADT and 4.04 (IQR 1.65, 9.91) for patients undergoing ADT alone. Irrespective of treatment modalities, cT4 patients had significantly lower survival rates compared to cT3 patients (OM: HR 1.44, IQR: 1.17,1.77; PCSM: HR 1.39 IQR: 1.06,1.82) (Table S1, Figure 1).

### 3.2 Radical surgery subgroup

A total of 27 primary cT4 PCa patients received pelvic surgery with radical intent as part of a multidisciplinary management and were included in the present study. Cystoprostatectomy was performed in 20 (74%) patients, six (22%) underwent cystoprostatectomy and rectum resection and one (4%) patient underwent prostatectomy and rectum resection.

Patients' demographic and clinical characteristics are shown in Table 2. The median follow‐up time was 5.7 ± 4.02 (0.4‐13.53) year. Most of the patients (81.5%) received neoadjuvant therapy; ADT alone was the most used NT (54.6%). The median time from diagnosis to surgical treatment in patients undergoing neoadjuvant therapy was 4.15 (IQR: 2.4, 5.9) months; 3 out of 22 (13.6%) patients who received NT showed local tumour growth or no size reduction at preoperative radiological evaluation. Surgical aspects and pathological findings are summarized in Table 2. Extended lymphadenectomy was performed in all cases. Following surgery, pathological stage pT4 was confirmed in 19 patients (70.4%), and rectum or pelvic wall invasion was demonstrated in five cases (18.5%). Positive pelvic lymph nodes (PLN) were detected in 20 patients (74.1%), 30% higher compared with preoperative examinations. Nine (33.3%) and one (3.7%) patient had Clavien‐Dindo III and IV complications within 90 days after surgery, respectively. Only three of them related to the upper urinary tract (ureteric anastomosis leakage or stenosis) (Table 3).

**TABLE 2:** Preoperative patients' characteristics and pathological findings after surgery.

Variable	Karolinska cohort
	*N* = 27
Age (year)	63 (60, 69)
PSA at diagnosis, ng/mL	31.0 (9.1, 70.0)
cN stage, *n* (%)	
cN0	16 (59%)
cN1	11 (41%)
cT Stage, *n* (%)	
cT4	27 (100%)
Bx ISUP GG, *n* (%)	
3	7 (25.9%)
4	4 (14.8%)
5	15 (55.6%)
Sarcomatoid	1 (3.7%)
Neoadjuvant therapy	
No	5 (18.5%)
Yes	22 (81.5%)
ADT	12 (54.5%)
RT/CH	2 (9.1%)
ADT + RT/CH	8 (36.4%)
Neoadjuvant therapy duration, months	4.15 (2.4, 5.9)
PSA at surgery, ng/mL	0.6 (0.2, 6.8)
pT Stage, *n* (%)	
pT3b	8 (29.6%)
pT4a	14 (51.9%)
pT4b	5 (18.5%)
Bladder invasion	19 (70.4%)
Rectum or pelvic wall invasion	5 (18.5%)
Nodes resected, *n* (%)	23.0 (17.0, 32.0)
pN stage, *n* (%)	
pN0	8 (29.6%)
pN1	19 (70.4%)
Surgical margins, *n* (%)	
Negative	20 (74.1%)
Positive	7 (25.9%)
Final ISUP GG, *n* (%)	
3	3 (11.1%)
4	3 (11.1%)
5	12 (44.4%)
Undifferentiated	9 (33.3%)

Abbreviations: ADT, androgen deprivation therapy; Bx ISUP GG, Gleason Grade group ISUP at diagnosis; CH, chemotherapy; PSA, prostate‐specific antigen; RT, radiotherapy.

**TABLE 3:** Surgical approach and complications within 90 days after surgery.

Variable	Karolinska cohort
	*N* = 27
Type of surgery, *n* (%)	
Prostatectomy + rectum resection	1 (3.7%)
Cystoprostatectomy	20 (74.1%)
Cystoprostatectomy + rectum resection	6 (22.2%)
Approach, *n* (%)	
Robotic	14 (51.9%)
Open	13 (48.1%)
90 Days complication, *n* (%)	
None	7 (25.9%)
Clavien 1	2 (7.4%)
Clavien 2	8 (29.6%)
Clavien 3	9 (33.3%)
Clavien 4	1 (3.7%)
Description of major complication (Clavien 3–4)	
Gastrointestinal	
Bowel obstruction	1 (3.7%)
Rectal fistula	1 (3.7%)
Infectious	
Pelvic abscess	1 (3.7%)
Sepsis/bacteremia	1 (3.7%)
Genitourinary	
Anastomosis urinary leakage (nephrostomy)	2 (7.4%)
Anastomosis stenosis	1 (3.7%)
Wound dehiscent surgery	2 (7.4%)
Others	
Compartmental syndrome	1 (3.7%)

**FIGURE 2:**
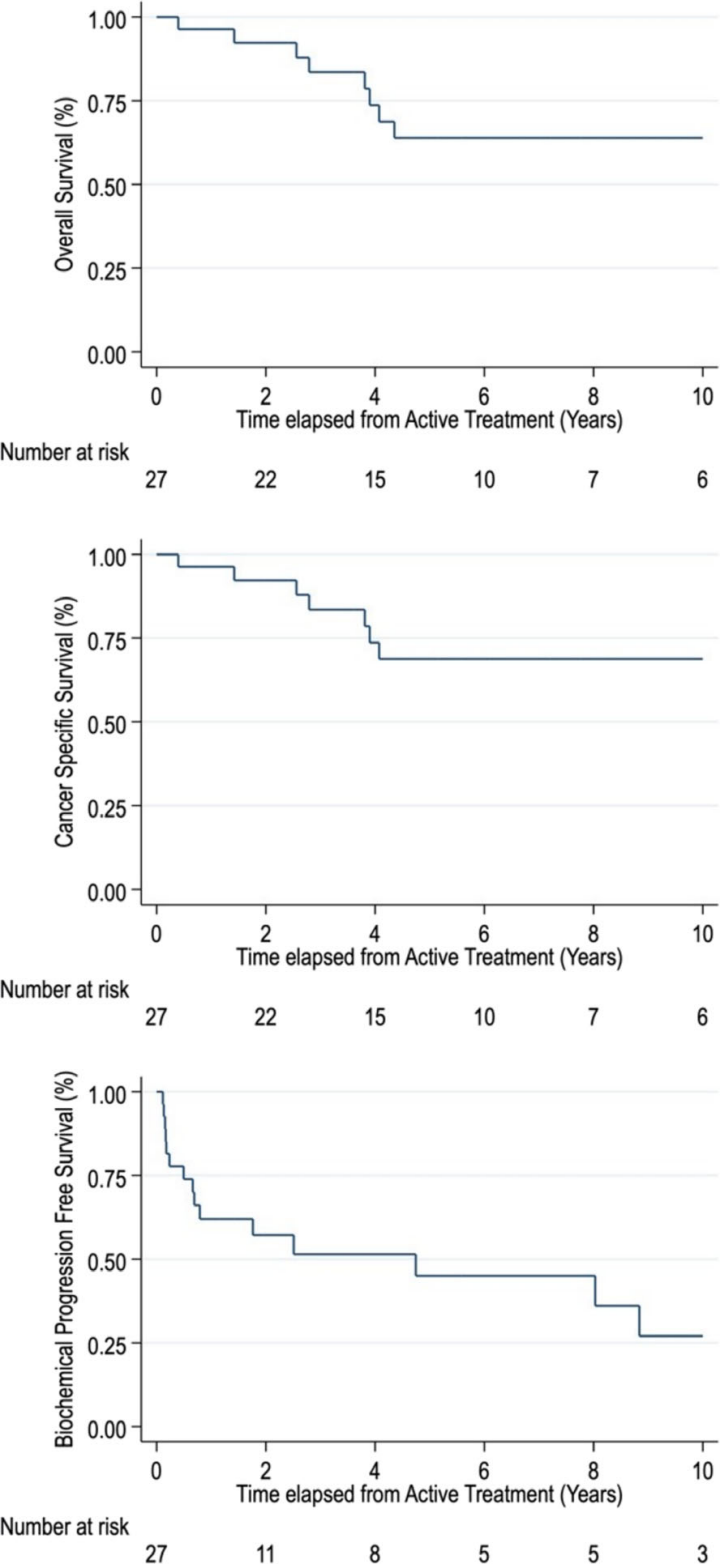
Overall survival, cancer‐specific survival and biochemical progression‐free survival of the Karolinska cohort.

Undetectable postoperative PSA was achieved by 21 patients (77.8%). Salvage treatment after biochemical or radiological progression was given to 19 patients (70.3%). One patient remained without neoadjuvant or adjuvant treatment until the end of the reviewed period. PSA recurrence was observed in 17 patients (62.9%), and median BPFS was 30 months. The projected 1‐ and 5‐year BPFS rate was 58.3% and 39.6%, respectively. Deaths associated with PCa were recorded in seven patients (25.9%). The CSS rate was at 1‐ and 5‐year, 96.3% and 68.8%, respectively, and the OS rate was 96.3% and 63.8%, respectively (Figure 2).

In the subgroup of patients who received NT, patients with radiological response to NT showed the best survival outcomes. The 5‐year CSS rate was 85.7% in NT responders compared to 100% of mortality at 5 years in the group of non‐NT responders.

## 4 DISCUSSION

Patients with a diagnosis of non‐metastatic LAPCa are at high risk to die from their disease. In the present study, we found that in the Stockholm region, the 10‐year CSS of patients with LAPCa ranged from 25% to 75% according to the initial treatment modality. In these patients and especially in patients with a diagnosis of cT4 disease, the most used treatment strategies were RT + ADT or ADT alone.

Similar results are reported from other population‐based studies. Hsiao et al. evaluated outcomes of 615 cT4N0M0 patients from the SEER database; 82 (13%) and 259 (42%) patients were treated with RP and RT, while 274 (45%) did not receive any form of local treatment. The 5‐ and 10‐year CSS rates were 71% and 60%.^9^ Akre et al. from the Swedish National PCa Register found similar treatment patterns suggesting undertreatment, particularly among men in older age groups. The 5‐year PCa mortality rate was 72% in patients treated with non‐curative intent.^10^

Two large phase III randomized clinical trials (RCT) demonstrated combined treatment of ADT plus RT showed a benefit in disease‐free survival, overall survival and PCa‐specific survival.^11^, ^12^ However, the demand for local tumour control to reduces the number of secondary surgeries due to local invasion emphasizes the need for the addition of radical surgical local treatment to a multimodal therapy. Indeed, emerging data support surgery in high‐risk disease with acceptable intermediate to long‐term oncological outcomes and low morbidity in well‐selected patients.

To prove the feasibility and efficacy of surgical treatment in patients with LAPCa, we described treatment protocols, outcomes and complications of our series of cT4 patients managed with pelvic surgery with radical intent due to the regional disease extension. Cystoprostatectomy or pelvic exenteration was deemed appropriate in this subset of patients to maximize radical resection and to not compromise curability. CSS at 10‐year was 68%, which is higher compared to previously reported outcomes after RT and ADT. Even if this represents outcomes of selected patients with cT4 patients managed in a multidisciplinary team with high level of expertise, other authors found similar survival rates (CSS >80%).^13^, ^14^ Notably, our cohort did not receive adjuvant treatment, and salvage treatment was administered in case of clinical recurrence.

NT may have played an important role in our results. Indeed, previous studies have shown that NT improves pathological outcomes, decreases the rate of positive surgical margins and lowers the incidence of positive lymph nodes. Up to one third of the patients in our cohort were down staged to pT3b, and we found that patients with radiological and biochemical response to the NT had excellent CSS and OS. To the best of our knowledge, radiological response after NT has never been tested as potential surrogate endpoint for survival in patients with PCa, and this may be an interesting point to address in future studies. It has been described that the greatest volume reduction occurred in the first 6 weeks^15^ and ranges between 10% and 54%.^16^ Not having a radiological response after NT can imply a very aggressive tumour with great potential for disease progression and therefore, very poor prognosis.

Historically, different schemes of NT have been proposed, including radiotherapy, androgen deprivation therapy or combined treatments, but due to controversial results^17^ and the potential risk of complications during or after surgery, its use has not extended.^18^, ^19^ A sub‐analysis in our results showed patients who received a combined NT, ADT plus either RT or chemotherapy had significant higher rates of progression‐free survival compared to the single NT group. However, it is a too small sample to draw a significant conclusion. In recent years, several clinical trials using combined approach such as RT and ADT,^20^ chemotherapy^21^ and next‐generation androgen receptor inhibitors have renewed the interest for NT in LAPCa patients and can impact significantly the management of these patients.

When performing radical cystoprostatectomy or pelvic exenteration for cT4 PCa, a risk of misclassification and overtreatment should be considered. In our study, 70.4% of the patients pT4 were confirmed, and 29.6% were down staged to pT3b. All of them have received NT and besides had a high grade (GS 9‐10 or sarcomatous differentiation) or pN1 (70%) disease implying poor prognosis. Many of these patients if not receiving a local treatment for PCa are likely to experience severe local symptoms, including lower urinary tract symptoms, haematuria, bilateral ureteric obstruction, postrenal insufficiency among others due to disease progression, frequently requiring an invasive procedure and compromising the patient's QoL.^22^, ^23^ Several authors have addressed the viability and safety of the palliative cystectomy in advanced PCa patients, demonstrating a low rate of complications, improving local control and providing effective palliation compared to other local procedures.^7^, ^24^ Considering most of LAPCa patients without distant metastases may have a life expectancy of more than 5 years, to choose a treatment that adequately achieve effective oncological and functional outcomes is a primary issue.

Our study provides some insight into the potential of cystoprostatectomy and/or pelvic exenteration as an option treatment for LAPCa. Surgery is proven to be technically feasible even in patients with extensive PCa disease with an acceptable rate of complications. One of the strengths of this study is the inclusion of patients who are not commonly selected for surgery and yet despite of the high mortality rate of pT4b and non‐NT responders, the rest of cohort have an acceptable survival outcome and an excellent local tumour control even in pT4b patients, as described by Leibovici et al.^23^ Nevertheless, surgery in this group of patients should be considered cautiously.

We acknowledged several limitations in this study. First, the selection bias implied by its retrospective design. The descriptive character of the analysis must be considered when interpreting the results. The tumour characteristics, age and performance status of the patients played an important role when selecting treatment type. Moreover, when analysing the second cohort from Karolinska hospital, the small sample precluded more in deep comparison within different approaches for NTs. Finally, we did not have information's on QoL questionaries, but this was behind the scope of the present report. Further large‐scale prospective studies should address this issue.

### 4.1 Conclusion

Non metastatic LAPCa is usually managed with RT and ADT with low CSS rates suggesting undertreatment in this subset of patients. Pelvic surgery with radical intent as part of a multidisciplinary management may be an effective alternative even in patients with cT4 PCa leading to local tumour control and an acceptable morbidity.

## AUTHOR CONTRIBUTIONS

*Conception and design*: N. Peter Wiklund, Abolfazl Hosseini, Fiorella L. Roldan, and Ugo Giovanni Falagario. *Acquisition of data*: Fiorella L. Roldan, Ugo Giovanni Falagario, Rodolfo Sánchez Salas, Markus Aly, and Lars Egevad. *Analysis and interpretation of data*: Fiorella L. Roldan, Ugo Giovanni Falagario, and N. Peter Wiklund. *Drafting of the manuscript*: Fiorella L. Roldan, Ugo Giovanni Falagario, N. Peter Wiklund, Anna Lantz, and Mats Olsson. *Critical revision of the manuscript for important intellectual content*: Anna Lantz, Olof Akre, Lars Egevad, and Mats Olsson. *Statistical analysis*: Ugo Giovanni Falagario. *Supervision*: N. Peter Wiklund, Abolfazl Hosseini, Henrik Grönberg, Olof Akre, and Anna Lantz.

## CONFLICT OF INTEREST STATEMENT

None of the contributing authors have any conflicts of interest, including specific financial interests and relationships and affiliations relevant to the subject matter or materials discussed in the manuscript.

## Supporting information


**Table S1.** Multivariable Cox regression analysis evaluating predictors of overall mortality and cancer specific mortality in patients diagnosed with non‐metastatic locally advanced Prostate cancer in the Stockholm region.
