# Supplementary material for: Long‐term oncological outcomes after multimodal treatment for locally advanced prostate cancer
Source: BJUI Compass. 2024 Aug 1;5(9):885–92. doi: 10.1002/bco2.414 (PMC11420104; doi:10.1002/bco2.414)
Supplement: Supplementary file 1 — Table S1. Multivariable Cox regression analysis evaluating predictors of overall mortality and cancer specific mortality in patients diagnosed with non‐metastatic locally advanced Prostate cancer in the Stockholm region. [file BCO2-5-885-s001.docx]

**Supplementary Table 1.** Multivariable Cox regression analysis evaluating predictors of overall mortality and cancer specific mortality in patients diagnosed with non-metastatic locally advanced Prostate cancer in the Stockholm region.

|  | **Overall Mortality** | | | **PCa Specific Mortality** | | |
| --- | --- | --- | --- | --- | --- | --- |
| **Covariate** | **HR** | **95% CI** | **P>\|z\|** | **HR** | **95% CI** | **P>\|z\|** |
| **Age at Diagnosis, per year** | 1.05 | 1.04,1.06 | <0.001 | 1.04 | 1.02,1.05 | <0.001 |
| **PSA, per ng/ml** | 1.00 | 1.00,1.00 | 0.119 | 1.00 | 1.00,1.00 | 0.026 |
| **CCI** |  |  |  |  |  |  |
| 0-1 | Ref. |  |  | Ref. |  |  |
| 2 | 0.37 | 0.29,0.46 | <0.001 | 0.11 | 0.06,0.20 | <0.001 |
| 3+ | 1.99 | 1.70,2.34 | <0.001 | 5.08 | 3.77,6.84 | <0.001 |
| **CT Stage** |  |  |  |  |  |  |
| cT3 | Ref. |  |  | Ref. |  |  |
| cT4 | 1.44 | 1.17,1.77 | 0.001 | 1.39 | 1.06,1.82 | 0.017 |
| **cN Staging** |  |  |  |  |  |  |
| cN0 | Ref. |  |  | Ref. |  |  |
| cN1 | 1.92 | 1.46,2.51 | <0.001 | 2.86 | 2.01,4.08 | <0.001 |
| cNX | 1.69 | 1.41,2.03 | <0.001 | 2.29 | 1.74,3.01 | <0.001 |
| **ISUP Grade** |  |  |  |  |  |  |
| 1* | Ref. |  |  | Ref. |  |  |
| 2 | 1.11 | 0.85,1.44 | 0.441 | 1.26 | 0.79,2.02 | 0.337 |
| 3 | 1.22 | 0.94,1.58 | 0.127 | 1.38 | 0.88,2.18 | 0.165 |
| 4 | 1.68 | 1.30,2.18 | <0.001 | 2.32 | 1.48,3.62 | <0.001 |
| 5 | 1.84 | 1.42,2.37 | <0.001 | 2.62 | 1.68,4.07 | <0.001 |
| **Treatment Year, per unit** | 0.99 | 0.98,1.01 | 0.558 | 0.97 | 0.94,0.99 | 0.019 |
| **Initial Treatment Modality** |  |  |  |  |  |  |
| RP | Ref. |  |  | Ref. |  |  |
| RT | 3.79 | 2.40,5.97 | <0.001 | 7.32 | 3.00,17.87 | <0.001 |
| Non-curative | 3.15 | 1.99,4.96 | <0.001 | 4.04 | 1.65,9.91 | 0.002 |
| **Salvage RT** |  |  |  |  |  |  |
| No | Ref. |  |  | Ref. |  |  |
| Yes | 1.87 | 0.96,3.64 | 0.064 | 1.84 | 0.53,6.44 | 0.338 |
